# The role of SorLA in neurotrophic activity and complex behavior

**DOI:** 10.1186/1750-1326-8-S1-P19

**Published:** 2013-09-13

**Authors:** Simon Glerup, Mart Saarma, Anders Nykjaer

**Affiliations:** 1 Biomedicine, Aarhus University, Aarhus, Denmark; 2 Institute of Biotechnology, University of Helsinki, Helsinki, Finland

## 

SorLA binds amyloid precursor protein (APP) in the trans-Golgi network and protects it against proteolysis by both the alpha- and beta-secretases, preventing the generation of toxic amyloid-beta peptide. We have recently found that SorLA also affects neuronal function in a different manner by acting as sorting receptor for the neurotrophic factor GDNF and its receptors GFRα1 and RET, directing them from the cell surface to endosomes. Through this mechanism, GDNF is targeted to lysosomes and degraded while GFRα1 and RET recycles, creating an efficient GDNF clearance pathway. SorLA thereby influences a number of neuronal systems known to depend on GDNF activity including the dopaminergic system and the hippocampus. Accordingly, mice with altered SorLA expression exhibit altered reward and memory function, and a behavioral phenotype characterized by altered anxiety, hyperactivity, and response to psychostimulants.

